# Germline Testing of Mismatch Repair Genes Is Needed in the Initial Evaluation of Patients With Muir-Torre Syndrome-Associated Cutaneous Sebaceous Neoplasms: A Case Series

**DOI:** 10.7759/cureus.33975

**Published:** 2023-01-19

**Authors:** Philip R Cohen, Razelle Kurzrock

**Affiliations:** 1 Dermatology, University of California, Davis Medical Center, Sacramento, USA; 2 Medicine, Medical College of Wisconsin Cancer Center and Genome Sciences and Precision Medicine Center, Milwaukee, USA; 3 Oncology, Worldwide Innovative Networking (WIN) Consortium, Villejuif, FRA

**Keywords:** somatic, sebaceous, neoplasm, muir-torre syndrome, mismatch repair, microsatellite instability, germline, epithelioma, carcinoma, adenoma

## Abstract

Muir-Torre syndrome, a subtype of Lynch syndrome, is characterized by a germline mutation of one or more mismatch repair genes such as MutL Homolog 1 (MLH1), MutS Homolog 2 (MSH2), MutS Homolog 6 (MSH6), and PMS1 Homolog 2, mismatch repair system component (PMS2) resulting in microsatellite instability and at least one malignancy and a minimum of one syndrome-associated sebaceous neoplasm such as a sebaceous adenoma, epithelioma, or carcinoma. The syndrome has an autosomal dominant mode of inheritance detectable with germline sequencing of normal body elements such as blood, saliva, or normal skin for a mismatch repair gene mutation. Sebaceous neoplasms can occur before, concurrent with, or following Muir-Torre syndrome-related cancer. Immunohistochemistry, microsatellite instability testing, and next-generation sequencing of tumor tissue can evaluate malignancies such as colorectal and endometrial cancer and sebaceous neoplasms for somatic mismatch repair gene defects. However, these tests cannot differentiate somatic (acquired) versus germline alterations, and immunohistochemistry and microsatellite stability assessment can produce false negatives. Finally, the Mayo Muir-Torre syndrome risk score algorithm cannot always reliably determine which patient with a new sebaceous neoplasm should have germline testing.

We report three men who presented with a Muir-Torre syndrome-associated sebaceous neoplasm: a 67-year-old male with no personal or family history of cancer who presented with a chest sebaceous carcinoma with MSH2 and MSH6 gene expression loss on immunohistochemistry and a Mayo Muir-Torre syndrome risk score of 0 who declined germline testing; a 74-year-old male with Janus kinase 2 (JAK2)-related myelodysplastic syndrome, yet no history of a Lynch syndrome-associated cancer, who developed a sebaceous epithelioma on his leg with PMS2 gene expression loss by immunohistochemistry and, although Mayo Muir-Torre syndrome risk score was only 1 (suggests no likelihood of a Lynch syndrome germline mismatch repair gene mutation), germline testing demonstrated a PMS2 alteration; and a 59-year-old male with a germline-confirmed MLH1-associated Lynch syndrome and a prior colon carcinoma, who developed a sebaceous adenoma on his nostril that unexpectedly demonstrated preservation of normal MLH1 staining (reflecting a false negative) by immunohistochemistry. In summary, these cases are consistent with the literature suggesting that tumor immunohistochemistry and microsatellite stability testing can miss germline alterations. Hence, we recommend that the initial evaluation of a patient with even a single new Muir-Torre syndrome-associated sebaceous neoplasm should include germline mismatch repair gene mutation testing. Finding a mismatch repair gene germline mutation should prompt genetic counseling, initial and future cancer screening recommendations, and germline testing of family members.

## Introduction

Muir-Torre syndrome is an autosomal dominant genodermatosis in which the patient develops not only a sebaceous neoplasm but also a malignancy. It is a subtype of hereditary nonpolyposis colorectal cancer, which is also known as Lynch syndrome. In addition to inherited genomic aberrations that are germline detectable, sporadic somatic mutations of the affected genes can also occur [1-3].

The sebaceous neoplasms associated with Muir-Torre syndrome include either an adenoma, an epithelioma, or a carcinoma. Patients often develop multiple sebaceous neoplasms and several visceral malignancies; however, even the detection of a single sebaceous neoplasm warrants the patient to be evaluated not only for the syndrome but also for cancer. The syndrome-associated cancer is most commonly gastrointestinal (such as colorectal cancer) or genitourinary (such as uterine cancer) [1,2,4,5].

Muir-Torre syndrome is caused by mutations of the deoxyribonucleic acid mismatch repair genes that correct errors during replication. The mismatch repair genes most associated with the syndrome include MutS Homolog 2 (MSH2) in 90% of patients and MutL Homolog 1 (MLH1) in approximately 10% of patients. MutS Homolog 6 (MSH6) and PMS1 Homolog 2, mismatch repair system component (PMS2) genes are less frequently observed [5-7].

Microsatellites are small repetitive nucleotide sequences in deoxyribonucleic acid. Microsatellite instability occurs when mutations in mismatch repair genes result in altered gene products and either insertions or deletions in the deoxyribonucleic acid microsatellites; this makes the microsatellites either abnormally long or short. Microsatellite instability initially causes mutations of oncogenes and tumor suppressor genes; subsequently, these result in carcinogenesis [8,9].

There are several methods to evaluate the presence of mismatch repair gene mutations. They utilize immunohistochemistry staining, microsatellite instability testing, and next-generation sequencing of the paraffin-embedded tumor. In addition, blood, saliva, or normal skin can be used for germline evaluation [3-20].

Three men who developed a sebaceous neoplasm are described. The sebaceous carcinoma of a previously cancer-free man showed loss of MSH2 and MSH6 staining; the diagnosis of Muir-Torre syndrome could not be established since his tumor specimen was not adequate for next-generation sequencing and he declined any further testing. Immunohistochemistry studies showed loss of PMS2 staining in the sebaceous epithelioma of the second man with myelodysplastic syndrome in whom Muir-Torre syndrome was subsequently confirmed by germline evaluation showing a PMS2 gene alteration. Finally, immunohistochemistry studies of a sebaceous neoplasm showed expression of all four mismatch repair genes (including MLH1, MSH2, MSH6, and PMS2) in the sebaceous adenoma of the third man with colon cancer and established Lynch syndrome (MLH1 germline alteration).

A diagnosis of inherited Muir-Torre syndrome warrants initial evaluation and life-long screening for syndrome-related malignancy [1,2,5]. In contrast to patients with an acquired, non-inherited, somatic mutation of a mismatch repair gene and a sebaceous neoplasm, an individual with inherited Muir-Torre syndrome has at least one syndrome-associated sebaceous neoplasm and a germline genetic aberration. Since the syndrome has an autosomal dominant inheritance pattern, evaluation of the patient’s parents, siblings, and children not only for the syndrome with germline testing but also as appropriate for screening for cancer should be performed. Therefore, it is reasonable to recommend that germline evaluation for mismatch replacement genes should be universally assessed as a primary evaluation for a patient who presents with a Muir-Torre syndrome-associated sebaceous neoplasm.

## Case presentation

Case 1

A 67-year-old male presented for evaluation of an asymptomatic acquired lesion on his central chest of three weeks duration, which he thought was an inflamed hair follicle. His past medical history is significant for actinic keratoses and allergic contact dermatitis to latex from Band-Aid adhesive. He has had no skin cancers or systemic malignancies. He has had a colonoscopy two years ago that only demonstrated benign polyps. He has no family history of cancer.

**Figure 1:**
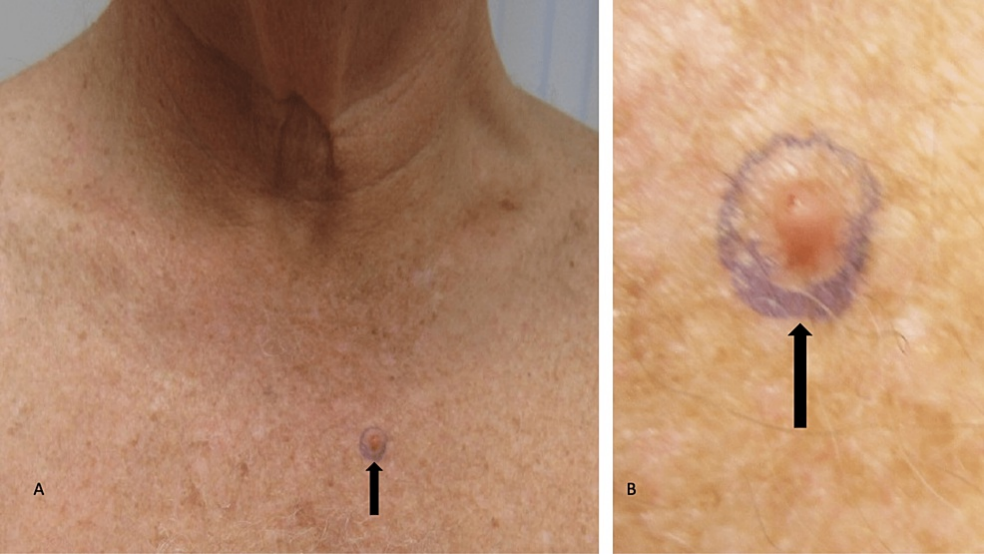
Cutaneous sebaceous carcinoma presenting as a red nodule on the chest of a 67-year-old male Distant (A) and closer (B) views of the left side of the upper chest show an acquired lesion of three weeks duration presenting as a nontender, firm, 5 × 5-millimeter, red, smooth nodule (black arrow). The man has no personal or family history of cancer. The purple-inked oval designates the borders of the shave biopsy that was performed.

Cutaneous examination showed a total of 20 keratotic plaques on his face, arms, and right leg; these were clinically consistent with actinic keratoses and treated with cryotherapy using liquid nitrogen. A 5 × 5-millimeter, red, smooth nodule was present on his upper chest, left of the midline (Figure 1); there were no palpable axillary lymph nodes. A shave biopsy was performed.

**Figure 2:**
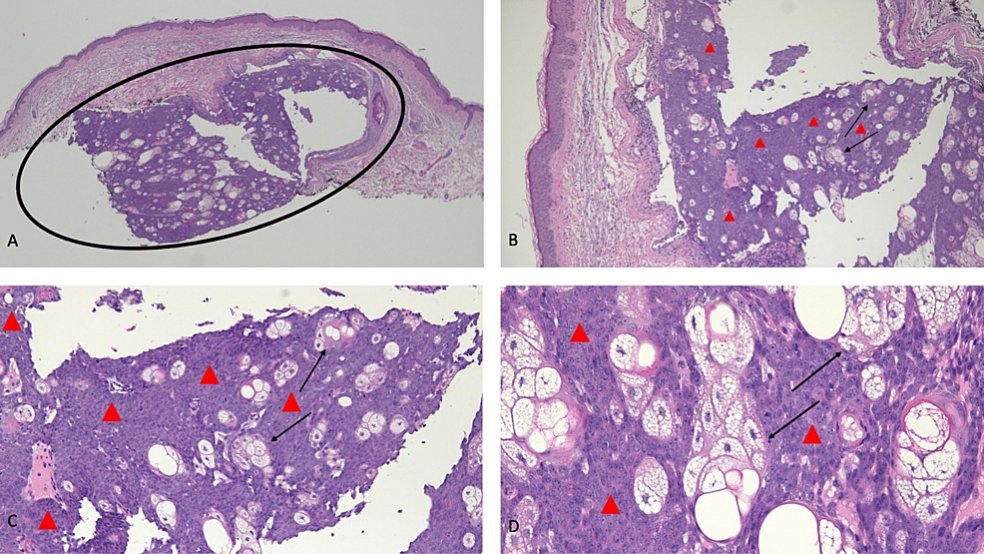
Pathologic presentation of an extraocular cutaneous sebaceous carcinoma on the chest of a 67-year-old male Distant (A) and closer (B, C, and D) views of the microscopic features of a sebaceous carcinoma show a basaloid nodule with prominent sebaceous differentiation in the dermis (within the black oval); the neoplasm appears to be extending from the squamous epithelium of a keratin-containing hair follicle located adjacent to the right side of the tumor. There are mature sebocytes (black arrows); however, approximately 90% of the tumor consists of crowded atypical-appearing basophilic-staining basaloid cells (red triangles) that have mitoses (hematoxylin and eosin stain: A, ×4; B, ×10; C, ×20; D, ×40).

Microscopic examination of the tissue specimen showed a basophilic-staining basaloid nodule with prominent sebaceous differentiation that did not demonstrate a definitive connection to the epidermis; however, there was surrounding squamous epithelium that appeared to be part of a follicle from which the dermal tumor may have branched from. The basaloid cells made up approximately 90% of the tumor; the cells were crowded with multiple mitoses including atypical mitotic figures and apoptotic bodies (Figure 2). The tumor extended to the deep margin of the specimen. The pathologic features established the diagnosis of a primary sebaceous carcinoma.

**Figure 3:**
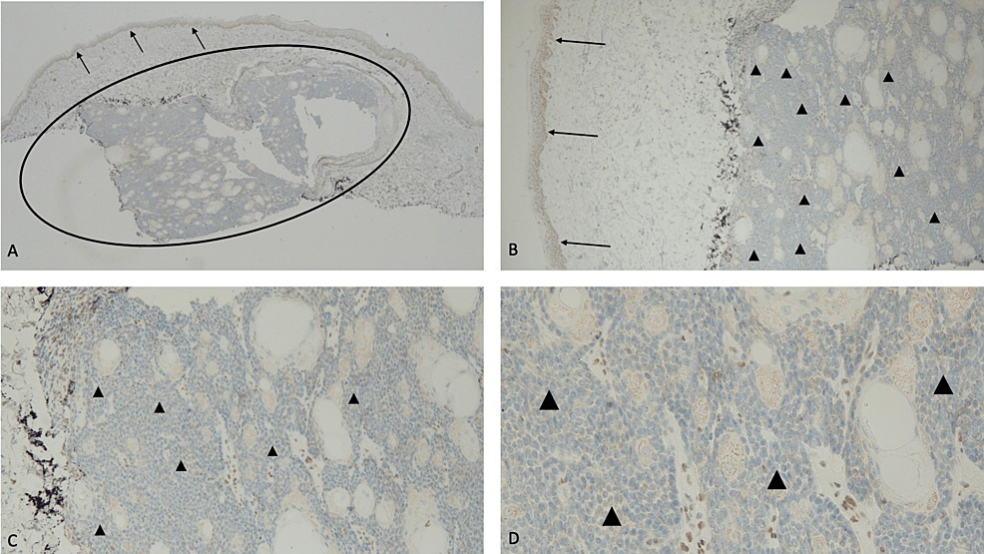
Immunohistochemistry analysis of sebaceous carcinoma for MSH2 gene expression shows an absence of staining Abbreviation: MSH2, MutS Homolog 2 Distant (A) and closer (B, C, and D) views of the tumor (within the black oval) show more than 90% loss of staining for MSH2. An absence of staining of the sebaceous carcinoma cells (black triangles) demonstrates a loss of expression of the MSH2 mismatch repair gene. Positive staining for MSH2 (black arrows) is present in the cells occupying the basal layers of the epidermis (immunoperoxidase stain: A, ×4; B, ×10; C, ×20; D, ×40).

**Figure 4:**
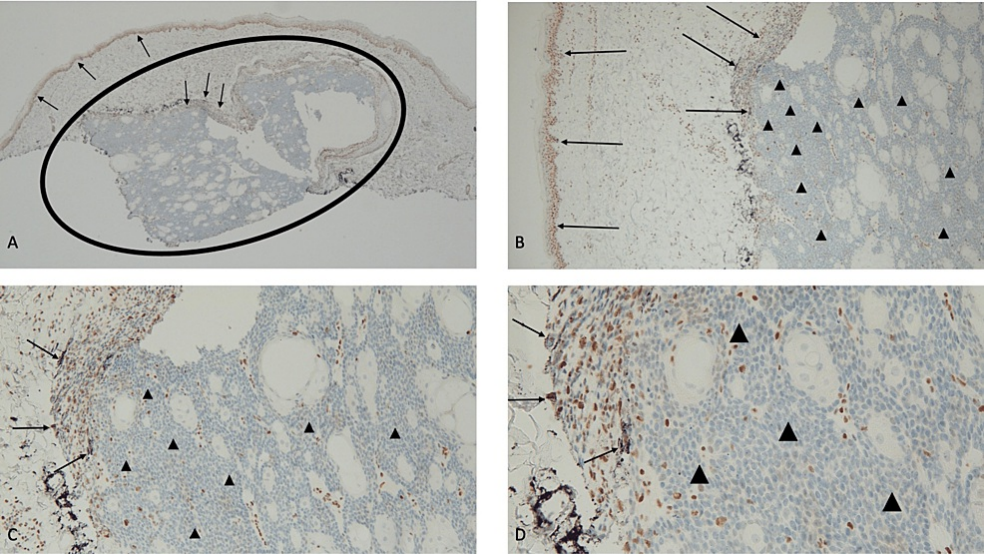
Immunohistochemistry analysis demonstrates an absence of staining for MSH6 gene expression in a sebaceous carcinoma Abbreviation: MSH6, MutS Homolog 6 Distant (A) and closer (B, C, and D) views demonstrate that there is a greater than 90% loss of staining for MSH6 in the sebaceous carcinoma (within the black oval). The basal layers of the epidermis and some of the cells at the periphery of the tumor show positive staining for MSH6 (black arrows). The absence of staining of the tumor cells (black triangles) shows a loss of expression of the MSH6 mismatch repair gene (immunoperoxidase stain: A, ×4; B, ×10; C, ×20; D, ×40).

**Figure 5:**
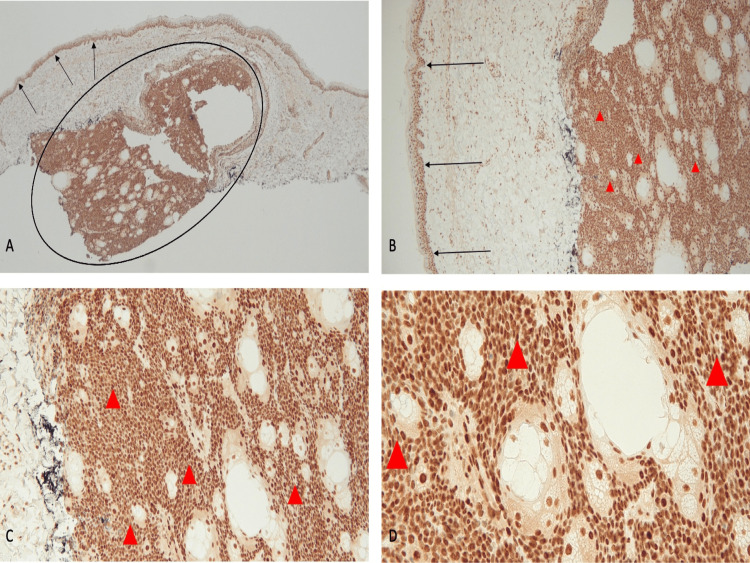
Staining for MLH1 gene expression is prominent in immunohistochemistry analysis of the sebaceous carcinoma Abbreviation: MLH1, MutL Homolog 1 Distant (A) and closer (B, C, and D) views of the tumor show strongly positive staining for MLH1 (within the black oval). The diffuse staining of the sebaceous carcinoma cells (red triangles) implies maintained expression of the MLH1 mismatch repair gene. Staining is also observed in the lower layers of the epidermis (black arrows) (immunoperoxidase stain: A, ×4; B, ×10; C, ×20; D, ×40).

**Figure 6:**
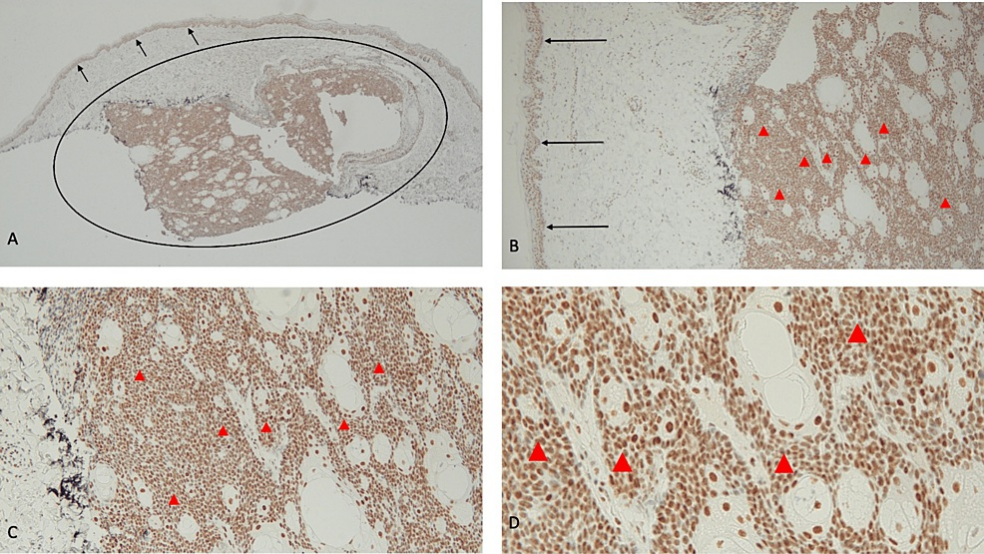
The sebaceous carcinoma shows staining for PMS2 when immunohistochemistry analysis was performed Abbreviation: PMS2, PMS1 Homolog 2, mismatch repair system component gene Distant (A) and closer (B, C, and D) views show strongly positive staining for PMS2 in the sebaceous carcinoma (within the black oval); the lower layers of the epidermis also show staining (black arrows). The maintained expression of the PMS2 mismatch repair gene is implied by the diffuse staining of the tumor cells (red triangles) (immunoperoxidase stain: A, ×4; B, ×10; C, ×20; D, ×40).

Immunohistochemistry staining was performed for MSH2, MSH6, MLH1, and PMS2. There was a greater than 90% loss of MSH2 (Figure 3) and MSH6 (Figure 4) within the tumor. However, there was retention of MLH1 (Figure 5) and PMS2 (Figure 6) within the dermal neoplasm. These findings were consistent with the possible loss of the MSH2 and the MSH6 mismatch repair genes.

Wide local excision of the tumor site was done by a surgical oncologist; there was no evidence of residual sebaceous carcinoma. A sentinel lymph node biopsy was also performed; there was no evidence of malignancy in the lymph node from the left axilla. The patient was also seen in consultation by a medical oncologist and a geneticist.

The sebaceous carcinoma was sent for next-generation sequencing; however, the specimen was insufficient for evaluation. Germline assessment was recommended by the oncologist; however, after meeting with the geneticist who emphasized his score of 0 using the Mayo Muir-Torre syndrome risk score algorithm (which includes variables such as age at presentation of initial sebaceous neoplasm, the total number of sebaceous neoplasms, personal history of Lynch-related cancer, and family history of Lynch-related cancers; patients with a score of 3 or more were more likely to have Muir-Torre syndrome, those with a score of 2 had intermediate likelihood, and no patient with a score of 0 or 1 was diagnosed with Muir-Torre syndrome (albeit with small numbers of patients in each category used in the development of the scoring system)), he declined any additional evaluation for himself or his family. He has not developed a recurrence of the sebaceous carcinoma nor systemic malignancy in eight years of follow-up.

Case 2

A 74-year-old male presented for evaluation of a new growing lesion on his left leg. He has previously been treated for actinic keratoses and several non-melanoma skin cancers including both basal cell carcinomas and squamous cell carcinomas. His past medical history was significant for a cholecystectomy and polycythemia vera of long duration; subsequently, he developed myelodysplastic syndrome. Initial germline testing demonstrated a Janus kinase 2 (JAK2) mutation (which may predispose to myeloid disorders); however, analysis for a PMS2 mutation had not been performed. He was regularly followed by a hematologic oncologist and was on a clinical trial receiving an oral JAK2 inhibitor daily.

He had no personal history of visceral malignancy; a sigmoidoscopy 13 years earlier and a colonoscopy two years earlier were both negative for polyps and cancer. His family history was significant for colon cancer (in a maternal uncle who died at age 67 years) and breast cancer (in his maternal grandmother who died at age 86 years). His son, at age 55 years, developed acute myelogenous leukemia; he successfully completed chemotherapy and a bone marrow transplant. His Mayo Muir-Torre syndrome risk score algorithm score was low (equal to 1).

**Figure 7:**
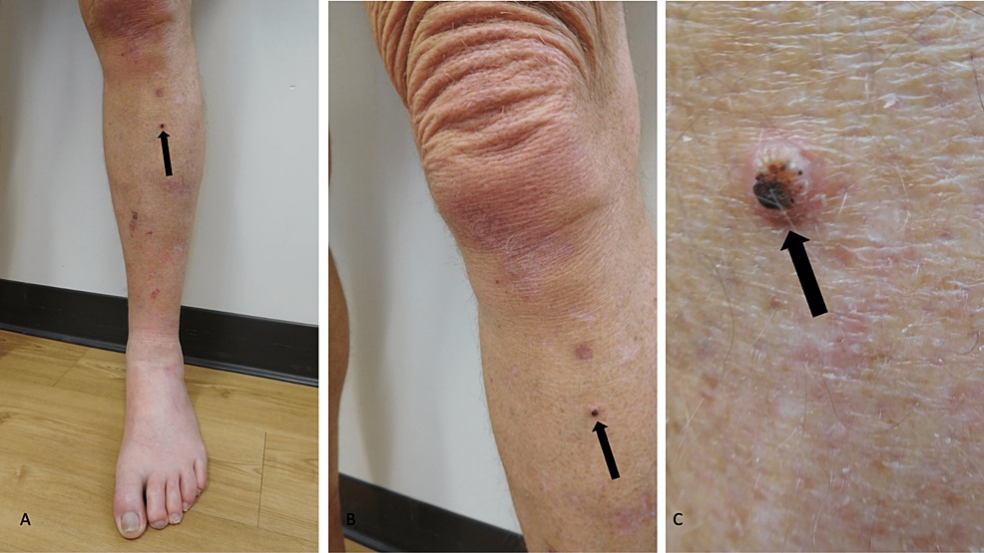
Sebaceous epithelioma presenting as a nodule on the lower left leg of a 74-year-old male Abbreviation: JAK2, Janus kinase 2 Distant (A) and closer (B and C) views of a new 8 × 8-millimeter, flesh-colored nodule (black arrow) on the left pretibial leg of a male with myelodysplastic syndrome who was receiving an oral JAK2 inhibitor. His family history was significant for colon cancer, breast cancer, and acute myelogenous leukemia; he had no personal history of visceral malignancy. A shave biopsy was performed.

Cutaneous examination showed several keratotic plaques on his face and distal arms; these were clinically consistent with actinic keratoses and treated with cryotherapy using liquid nitrogen. An 8 × 8-millimeter, partially crusted, flesh-colored nodule was present distal to his knee on the left anterior pretibial leg (Figure 7); there were no palpable inguinal lymph nodes. A shave biopsy was performed.

**Figure 8:**
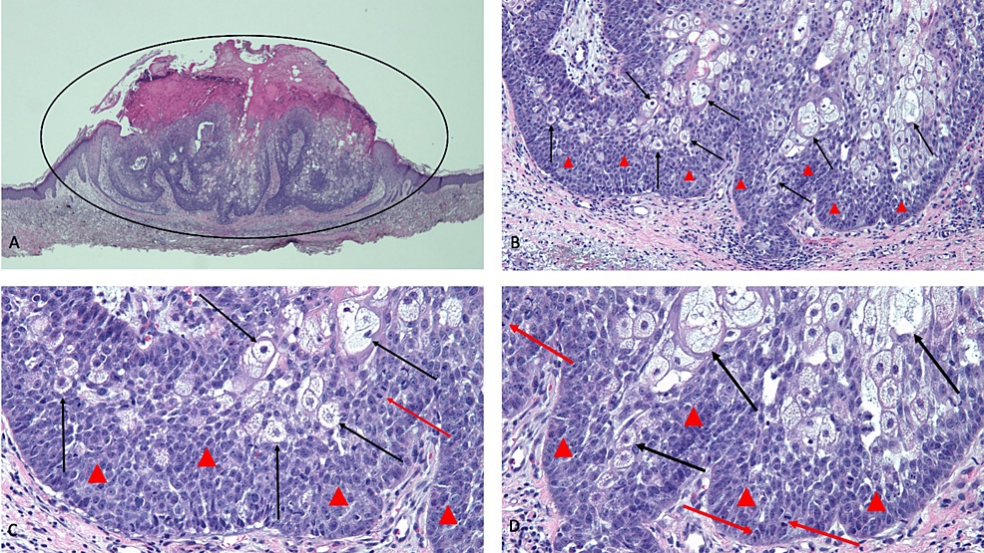
Pathologic presentation of an extraocular cutaneous sebaceous carcinoma on the chest of a 74-year-old male Distant (A) and closer (B, C, and D) views of the microscopic features of a sebaceous epithelioma show a tumor (within the black oval) that consists of numerous sebaceous lobules extending from the epidermis into the dermis; hemorrhagic crust overlies the epidermis. The tumor consists predominantly of basophilic germinative cells (red triangles) admixed with more mature sebaceous cells (black arrows). Atypical mitotic figures, within some of the tumor cells, are also observed (red arrows) (hematoxylin and eosin stain: A, ×4; B, ×10; C, ×20; D, ×40).

Microscopic examination of the tissue specimen showed a hemorrhagic crust overlying the epidermis. Extending from the epidermis into the dermis is a proliferation of numerous sebaceous lobules composed primarily of basophilic germinative cells admixed with more mature sebaceous cells. Mitotic figures, some of which are atypical, were also noted. In the planes of the sections examined, the lateral margins and the base of the specimen were not involved by the tumor (Figure 8).

The diagnosis was an atypical sebaceous epithelioma; this designation of atypical was based on the presence of atypical mitoses. Indeed, the pathologist commented that while the pathologic features were not absolutely diagnostic of malignancy, a low-grade sebaceous carcinoma may appear similar. Therefore, a wide local excision of the tumor site was performed.

**Figure 9:**
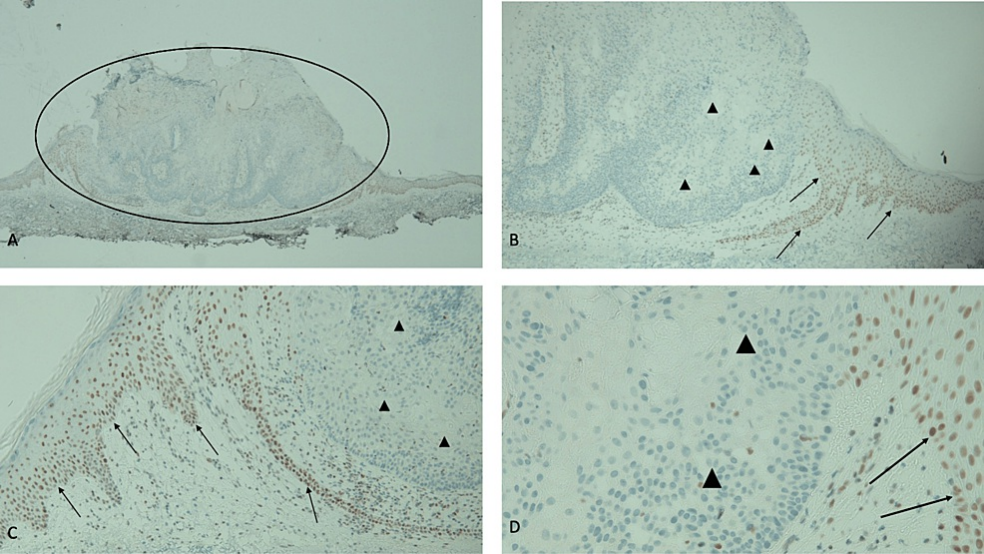
Immunohistochemistry analysis of sebaceous epithelioma for PMS2 expression shows an absence of staining Abbreviation: PMS2, PMS1 Homolog 2, mismatch repair system component gene Distant (A) and closer (B, C, and D) views of the tumor (within the black oval) show a complete loss of staining for PMS2. An absence of staining of the sebaceous epithelioma cells (black triangles) demonstrates a loss of expression of the PMS2 mismatch repair gene. Positive staining for PMS2 (black arrows) is present in the normal epidermis adjacent to the tumor (immunoperoxidase stain: A, ×4; B, ×10; C, ×20; D, ×40).

**Figure 10:**
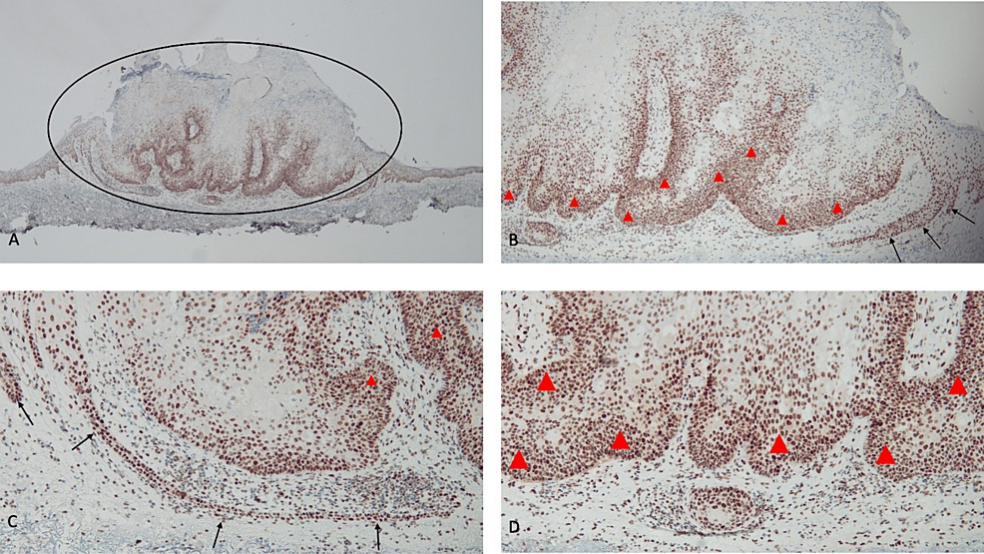
Staining for MLH1 gene expression is prominent in the sebaceous epithelioma when immunohistochemistry analysis was performed Abbreviation: MLH1, MutL Homolog 1 Distant (A) and closer (B, C, and D) views of the tumor show strongly positive staining for MLH1 (within the black oval). The diffuse staining of the lower portion of the sebaceous epithelioma cells (red triangles) implies maintained expression of the MLH1 mismatch repair gene. Staining for MLH1 is also observed in the normal adjacent epidermis (black arrows) (immunoperoxidase stain: A, ×4; B, ×10; C, ×20; D, ×40).

**Figure 11:**
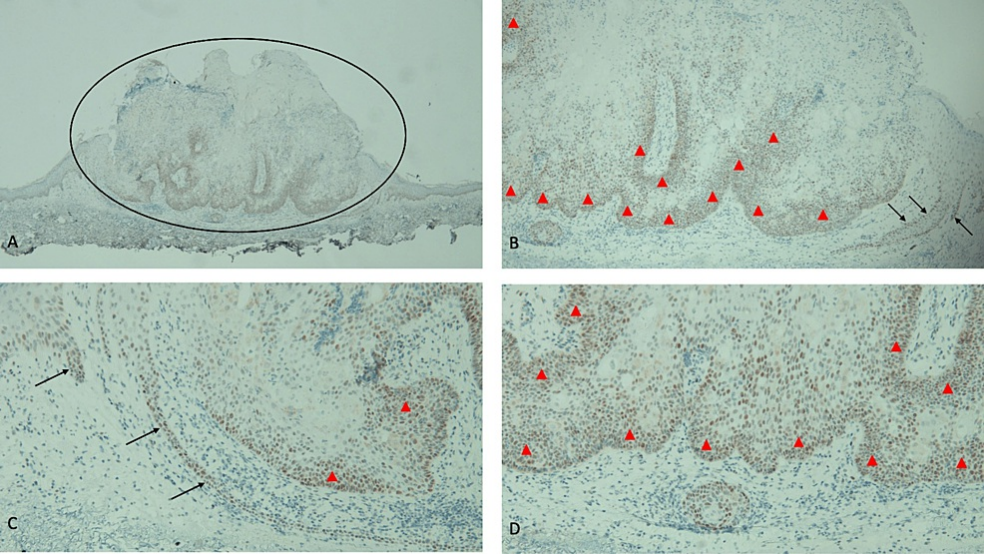
Immunohistochemistry analysis of sebaceous epithelioma for MSH2 gene expression shows staining Abbreviation: MSH2, MutS Homolog 2 Distant (A) and closer (B, C, and D) views of the lower portion of the tumor (within the black oval) show staining for MSH2. Strongly positive staining of the cells in the lower portion of the sebaceous epithelioma cells (red triangles) demonstrates preservation of MSH2 mismatch repair gene expression. Positive staining for MSH2 (black arrows) is also noted in the normal epidermis adjacent to the tumor (immunoperoxidase stain: A, ×4; B, ×10; C, ×20; D, ×40).

**Figure 12:**
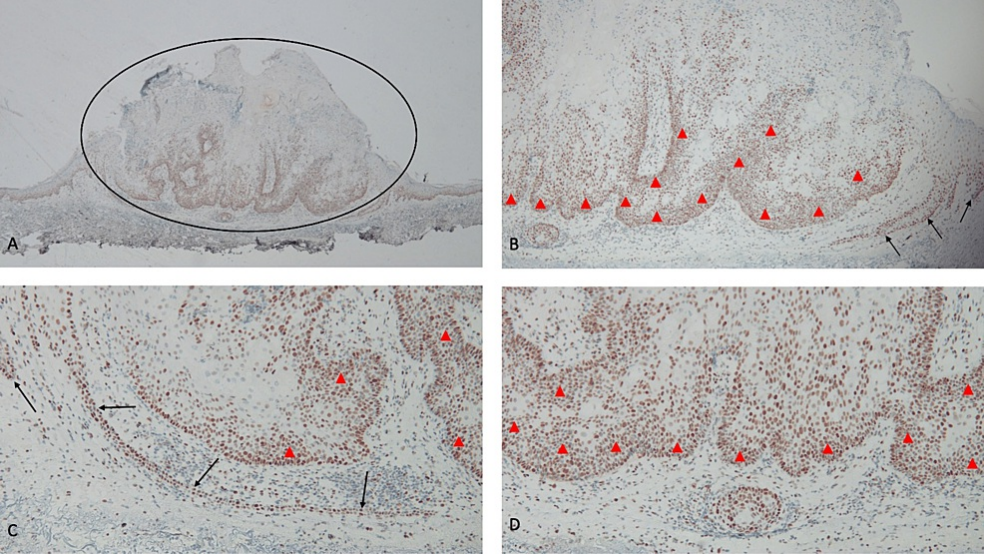
Immunohistochemistry analysis demonstrates staining for MSH6 gene expression in a sebaceous epithelioma Abbreviation: MSH6, MutS Homolog 6 Distant (A) and closer (B, C, and D) views demonstrate staining for MSH6 in the lower portion of the sebaceous epithelioma (within the black oval). The epidermis adjacent to the tumor also shows positive staining for MSH6 (black arrows). The staining of the tumor cells (black triangles) shows that the expression of the MSH6 mismatch repair gene is maintained (immunoperoxidase stain: A, ×4; B, ×10; C, ×20; D, ×40).

Immunohistochemistry staining was performed for MSH2, MSH6, MLH1, and PMS2. There was a complete loss of PMS2 staining (Figure 9). There was no loss of staining with MLH1 (Figure 10), MSH2 (Figure 11), and MSH6 (Figure 12). These findings were consistent with the possible loss of mismatch repair genes.

Based on the loss of PMS2 staining within his sebaceous skin neoplasm, a colonoscopy was performed. An adenomatous polyp with high-grade dysplasia was removed. Subsequent immunohistochemical studies demonstrated an absence of PMS2 staining.

He was evaluated by a geneticist. Germline testing specifically for PMS2 was performed. A skin biopsy, transported on saline, was provided so that fibroblasts could be grown and tested since a blood, buccal, or saliva sample would not be adequate for the evaluation because of his myelofibrosis. The familial pathogenic PMS2 gene variant, involving the nucleic acid change c.1939A>T, was detected; the specific amino acid alteration p.Lys647Ter was detected by targeted sequencing.

A diagnosis of PMS2-associated Muir-Torre syndrome was established. This was based on the presence of his sebaceous neoplasm and his systemic malignancies, which included not only his polycythemia vera and myelodysplastic syndrome but also the adenomatous polyp with high-grade dysplasia. After receiving his approval, the geneticist informed his four children of the diagnosis, recommendation for cancer screening, and possible additional genetic evaluation; his son who had acute myelogenous leukemia also had a germline mutation of his PMS2 gene.

The patient regularly followed up with his hematologic oncologist and dermatologist. He did not develop any additional sebaceous neoplasm. Another colonoscopy was scheduled to be performed in two years; however, he died from myelodysplastic syndrome-related complications prior to the planned procedure.

Case 3

A 59-year-old male presented for evaluation of a lesion on his nose. The small bump had been present for four years. It would increase and decrease in size. It would become crusted and then heal.

He also had a history of actinic keratoses, which had been treated with topical 5-fluorouracil cream or cryotherapy using liquid nitrogen. He had neither non-melanoma skin cancer nor melanoma. He had facial seborrheic dermatitis that was successfully managed topically with desonide 0.05% cream and ketoconazole 2% cream; he would also use ketoconazole 2% shampoo on his face.

His past medical history was significant for cervical stenosis of the spine, diverticulosis, gastroesophageal reflux disease, hyperlipidemia, hypertension, hydrocele, and a transient ischemic attack. His family history was significant for both of his parents and his brother having cancer. His mother had primary colorectal cancer twice and cancer of her pancreas and liver. His father had liver cancer, and his brother had kidney and rectal cancer.

He was diagnosed with Lynch syndrome 16 years ago, at age 43 years. He requested germline evaluation based on the multiple cancers developed by his mother. His testing revealed an MLH1 germline mutation characterized by R226X premature truncation.

Ten years later, at age 53 years, a screening colonoscopy detected an intramucosal adenocarcinoma (pTis) arising focally in a tubular adenoma located in his ascending colon. The adenoma had been completely resected during the biopsy. He elected for surveillance rather than surgery; therefore, the site was marked with a tattoo and he has a colonoscopy every six months to one year. There has been no recurrence.

**Figure 13:**
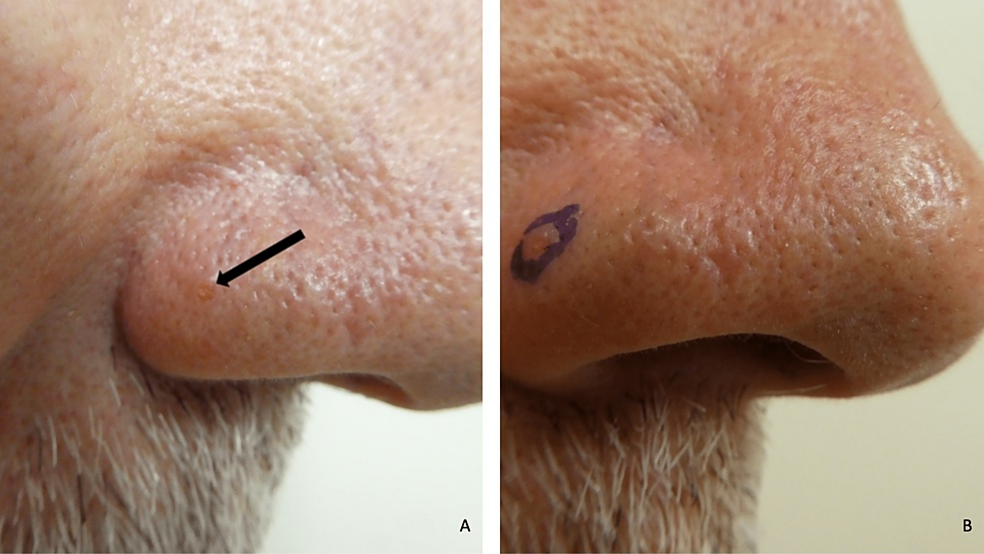
Sebaceous adenoma presenting as a papule on the nose of a 59-year-old male Abbreviation: MLH1, MutL Homolog 1 Distant (A) and closer (B) views of the right nostril show a lesion of four years duration appearing as a 1 × 1-millimeter, flesh-colored, smooth papule with a central umbilication on the right nostril (black arrow and within the purple-inked oval). The man had germline-confirmed MLH1-associated Lynch syndrome; he had an intramucosal adenocarcinoma (pTis) arising focally in a tubular adenoma located in his ascending colon diagnosed at age 53 years. His family history was significant for colorectal cancer (mother had two primary neoplasms), kidney cancer (brother), liver cancer (mother and father), pancreatic cancer (mother), and rectal cancer (brother). The diagnosis of MLH1-associated Muir-Torre syndrome was established after the microscopic evaluation of the lesion, which was completely removed during the punch biopsy using a 2-millimeter biopsy instrument, showed a sebaceous adenoma; immunohistochemistry analysis of the sebaceous tumor unexpectedly showed preservation of the MLH1 mismatch repair gene.

Cutaneous examination of his face showed a 1 × 1-millimeter, flesh-colored, smooth papule with a central umbilication on the right nostril (Figure 13). The clinical differential diagnosis included a fibrous papule, basal cell carcinoma, and sebaceous neoplasm, such as an adenoma or epithelioma. A punch biopsy, using a 2-millimeter biopsy instrument attempting to remove the entire lesion, was performed.

Microscopic examination of the tissue specimen showed a cup-shaped depression with basaloid sebocytes lining the upper segment of a dilated follicle. The sebocytes were focally crowded but lacked atypia. Maturation toward the center of the individual sebaceous lobules was apparent. The basaloid cells accounted for less than 50% composition of the tumor. The diagnosis was a sebaceous adenoma; the biopsy completely removed the benign neoplasm.

Immunohistochemistry staining was performed for MLH1, MSH2, MSH6, and PMS2 mismatch repair gene expression. There was no loss of staining for all four of the immunostains. All stains showed avid expression; hence, the presence of MLH1 staining did not correlate with his preexisting germline-confirmed diagnosis of MLH1-associated Lynch syndrome.

A diagnosis of MLH1-associated Muir-Torre syndrome was established. This was based on the presence of his colon cancer that preceded the development of his sebaceous adenoma by six years. He had previously been evaluated by a geneticist; he has no children, and the remainder of his family have been evaluated for Lynch syndrome and have periodic cancer screenings. He continues to be followed not only by his internist and dermatologist but also by a gastroenterologist and medical oncologist. His follow-up cancer screening consists of colonoscopy every one to two years, capsule endoscopy every three years, annual urine cytology, dermatology skin examination every six months, and the following evaluations annually: pancreatic imaging (alternating with an endoscopic ultrasound and a magnetic resonance imaging every other year), serum carcinoembryonic antigen level, and urine cytology.

## Discussion

Muir-Torre syndrome

Muir et al. reported a Maltese man who had a single sebaceous adenoma on his face, multiple facial keratoacanthomas, and seven primary malignant solid tumors in March 1967. The patient had previously been presented by Dr. Smith at the meeting of the Dermatologic Section of the Royal Society of Medicine nine years earlier. Subsequently, in October 1967, Torre presented (and subsequently published) a man with more than 100 sebaceous neoplasms and primary carcinomas of the ampulla of Vater and the colon at the meeting of the New York Dermatologic Society. The association of sebaceous neoplasms and visceral malignancies was recognized as a subtype of Lynch syndrome in 1981; the eponym Muir-Torre syndrome was proposed the following year in 1982 [2].

Muir-Torre syndrome is a subtype of Lynch syndrome. Pathologists currently evaluate all colorectal carcinomas and endometrial carcinomas with not only immunohistochemistry testing for mismatch repair gene mutation but also polymerase chain reaction studies for microsatellite instability to determine if the patient has Lynch syndrome. If either test is positive, referral for genetic consultation and germline evaluation is usually conducted [1,2,8].

The detection of a Muir-Torre syndrome-associated sebaceous neoplasm presents important issues regarding not only the evaluation of the patient for Muir-Torre syndrome but also whether the patient and their family should be evaluated for malignancy [5-7]. Over 30 years ago, in 1991, we performed a world literature review of all published cases of Muir-Torre syndrome and proposed a cancer workup for the patient and the patient’s family based on the malignancies most frequently associated with the syndrome and the American Cancer Society recommendations for cancer detection in asymptomatic people [1]. However, at that time, cancer-related immunohistochemistry for these types of patients had only recently been established; the association of Muir-Torre syndrome and mismatch repair genes had not been elucidated, and evaluation techniques such as microsatellite instability, next-generation sequencing, and germline testing had not been discovered. Since our earlier paper, there have been many significant advances in the evaluation of cancer-related syndromes and the treatment of their associated systemic malignancies.

Immunohistochemistry analysis

Antibodies bind to specific antigens. Monoclonal antibodies have been developed for use on formalin-fixed paraffin-embedded tissue. Based on these diagnostic advances, immunohistochemistry analysis is now commonly performed in pathology laboratories to characterize the tissue specimen beyond the morphologic features observed after routine staining. An aberrant immunohistochemistry result to mismatch repair proteins, manifested by a marked decrease or absence of staining for the antibody, indicates a loss of gene expression and therefore a potential genomic mutation [8,11].

Immunohistochemistry analysis of the mismatch repair genes MLH1, MSH2, MSH6, and PMS2 is regularly evaluated on colorectal cancers and endometrial carcinomas to screen for Lynch syndrome [8]. In addition, immunohistochemistry testing for these same genes has frequently been done on Muir-Torre syndrome-associated skin sebaceous neoplasms; indeed, one group of researchers have been able to correlate pathologic features of the tumors with the mismatch repair gene staining pattern [16]. The discovery of gene expression loss by an immunohistochemistry result showing diminished or absent staining for one or more of these genes on Muir-Torre syndrome-associated skin sebaceous neoplasms has prompted several investigators to recommend additional evaluation of the patient and their family for genetic counseling or cancer screening or both [5,6,8,11].

Discordant results for immunohistochemistry analysis (preserved expression of MLH1) and germline testing (MLH1 alteration) results were observed for our 59-year-old man in this report with an established germline mutation-confirmed MLH1-associated Lynch syndrome whose sebaceous adenoma has preservation of MLH1 staining (Table 1). In addition, researchers have assessed the predictive value of immunohistochemistry screening results of skin sebaceous neoplasm with regard to identifying Lynch syndrome patients; they have compared immunohistochemistry analysis with Lynch syndrome-confirmed diagnosis by germline mutation evaluation (Table 2) [10,12,13,18,19]. However, each of these studies has documented several patients with preserved expression of mismatch repair genes and germline documentation of mismatch repair gene mutation; if only immunohistochemistry analysis had been performed, the diagnosis of a mutation in the mismatch repair genes would not have been detected, and these individuals and their families would not have been evaluated for cancer and the possible diagnosis of Muir-Torre syndrome or Lynch syndrome.

**Table 1:** Correlation of immunohistochemistry studies and germline mutations for the men in this report Abbreviations: IHC, immunohistochemistry study; MLH1, MutL Homolog 1 gene; MSH2, MutS Homolog 2; MSH6, MutS Homolog 6; PMS2, PMS1 Homolog 2, mismatch repair system component gene ^a^IHC was performed using stains to evaluate for mismatch repair gene expression of MLH1, MSH2, MSH6, and PMS2. ^b^There was a lack of staining for MSH2 and MSH6; staining was demonstrated for MLH1 and PMS2. ^c^There was a lack of staining for PMS2; staining was demonstrated for MLH1, MSH2, and MSH6. ^d^Staining was demonstrated for MLH1, MSH2, MSH6, and PMS2.

Case	Sebaceous neoplasm diagnosis age (years)	Sebaceous neoplasm	IHC staining results (lack of staining showing an absence of gene expression)^a^	Germline mutation	Correlation of IHC and germline mutation
1	67	Carcinoma	MSH2, MSH6^b^	Testing not done	Unknown
2	74	Epithelioma	PMS2^c^	PMS2	Yes
3	59	Adenoma	Negative^d^	MLH1	No

**Table 2:** Studies assessing immunohistochemistry analysis and germline mutation evaluation in patients with sebaceous neoplasms: data and author’s conclusions Abbreviations: GlM, germline mutation; IHC, immunohistochemistry; LS, Lynch syndrome; MMR, mismatch repair; MSI, microsatellite instability; MTS, Muir-Torre syndrome; pts, patients; SN, sebaceous neoplasm; +, positive (abnormal test result); #, number of ^a^# MTS pts with + IHC/# MTS pts studied (% +). ^b^# non-MTS pts with + IHC/non-MTS pts studied (% +). ^c^# pts with SN and + IHC/# all pts with SN studied (% +). ^d^# pts with + MTS/# + IHC pts tested (% +). ^e^# pts with GlM for MMR genes /# + IHC pts (% +). ^f^# pts without GlM/# + IHC pts (% +). ^g^# pts with + IHC/# SN pts with IHC testing (% +). ^h^# pts with + GlM for MMR genes/# SN pts in the study (% +). ^i^# SN with + IHC/# SN with IHC testing (% +). ^j^# pts with + IHC/# SN pts with IHC testing (% +). ^k^# pts with + GlM for MMR genes/# SN pts in the study (% +). ^l^# lesions with + IHC/# lesions with IHC testing in LS pts (% +). ^m^# pts with molecular confirmation of + GlM for MMR genes/# LS pts evaluated with germline genetic testing (% +). ^n^# pts with + IHC/# SN pts with IHC testing who were evaluated by genetics and underwent genetic testing (% +). ^o^# pts with molecular confirmation of + GlM for LS/# SN pts who were evaluated by genetics and underwent genetic testing (% +).

Author	Year	IHC+ results	GlM+ results	Author’s conclusions	Reference
Roberts et al.	2013	11/13 (85)^a^, 40/77 (52)^b^, 51/90 (57)^c^	8/14 (57)^d^	“Germline genetic testing is the standard for diagnosing MTS.”	[10]
Everett et al.	2014	14/38 (37)^e^, 24/38 (63)^f^, 38/77 (49)^g^	25/86 (29)^h^	“Clinical genetics evaluation is warranted for patients with abnormal IHC test results, normal IHC test results with personal or family history of other LS-associated neoplasms, and/or multiple sebaceous neoplasms.”	[12]
Nguyen et al.	2020	36/38 (95)^i^, 10/11 (91)^j^	11/11 (100)^k^	“In patients with MTS, there is high intrapatient concordance of MMR protein IHC between different sebaceous neoplasms and with the patient’s underlying germline mutations, particularly in lesions on extrafacial sites.”	[19]
Aziz et al.	2022	239/253 (94.5)^l^	380/432 (88)^m^	“IHC and/or MSI testing of skin tumors in patients with a family history of LS-associated cancers may be a useful approach in identifying patients requiring referral to clinical genetics and/or consideration of germline genetic testing for LS.”	[18]
Kattapuram et al.	2022	44/61 (72)^n^	8/45 (18)^o^	“Cancer genetics referral of patients with sebaceous lesions, particularly for lesions with abnormal IHC, yields a significant rate of LS diagnosis. Providers should consider genetics referral for patients with sebaceous lesions.”	[13]

Roberts et al. conducted a retrospective study of 90 patients with sebaceous neoplasms (including sebaceous adenoma, sebaceous epithelioma, sebaceous carcinoma, and basal cell carcinoma or squamous cell carcinoma with sebaceous differentiation) who underwent mismatch repair immunohistochemistry analysis (Table 2). Loss of expression of mismatch repair genes occurred in 40 of 77 (52%) non-Muir-Torre syndrome patients. However, only 11 of 13 (85%) patients with known or suspected Muir-Torre syndrome had an abnormal mismatch repair gene immunohistochemistry result; therefore, two (15%) of these patients’ sebaceous neoplasms had preservation of mismatch repair protein expression. Fourteen of the patients with an abnormal immunohistochemistry result completed germline testing; eight (57%) had a germline mismatch repair gene mutation [10].

Everett et al. performed a retrospective study of 86 patients with sebaceous neoplasm (including sebaceous adenoma, sebaceous epithelioma, sebaceoma, and undescribed sebaceous neoplasm) who were referred for a clinical genetic evaluation (Table 2); only 25 of these individuals had a germline mutation. Immunohistochemistry analysis was done in 77 patients; only 14 of the 38 (37%) patients with positive immunohistochemistry results had a germline mismatch repair gene mutation. Importantly, similar to the man with sebaceous adenoma in this report, 11 of the 25 (44%) patients with germline confirmed mutation did not have loss of mismatch repair gene expression on immunohistochemistry analysis [12].

Nguyen et al. also did a retrospective study of 11 germline testing-confirmed Muir-Torre syndrome patients who each had two or more sebaceous neoplasm; they evaluated 38 tumors including 28 sebaceous adenomas, four sebaceous epitheliomas, and six sebaceous neoplasms, not otherwise specified (Table 2). They performed immunohistochemistry analysis of the 38 sebaceous neoplasms from the 11 patients; two sebaceous adenomas, accounting for 5% of the 38 sebaceous neoplasms, in a single Muir-Torre syndrome patient did not show loss of mismatch repair gene expression. Hence, again similar to the man with sebaceous adenoma in this report, the negative immunohistochemistry analysis from one of 11 (9%) patients did not correlate with his germline mutation-established Muir-Torre syndrome [19].

Aziz et al. recently did a systematic literature review to evaluate the cutaneous manifestation of molecularly confirmed Lynch syndrome patients (Table 2). Skin lesions included sebaceous adenoma, sebaceous epithelioma, sebaceous carcinoma, keratoacanthoma, basal cell carcinoma, squamous cell carcinoma, melanoma, and other tumors. Germline mutation testing had been performed in 432 patients; it was positive in 380 individuals. Immunohistochemistry analysis had been done in 253 skin lesions from patients with germline mutation-confirmed Lynch syndrome. Fourteen (5.5%) skin lesions from Lynch syndrome patients were mismatch repair gene proficient; there was no loss of mismatch repair gene expression on immunohistochemistry staining for five sebaceous adenomas (2% of skin lesions) and nine (3.5%) other lesions that included two keratoacanthomas, two squamous cell carcinomas, one melanoma, and four other tumors [18].

Kattapuram et al. also recently evaluated 447 patients with 473 sebaceous neoplasms including sebaceous carcinoma, sebaceous adenoma, sebaceoma, and sebaceous neoplasms, not otherwise specified, in a retrospective study (Table 2). A genetics referral was recommended for 98 patients; however, only 63 patients attended the appointment. Of the 63 patients seen by genetics, 61 patients had prior immunohistochemistry studies of their sebaceous neoplasm that were abnormal in 44 (72%) patients and normal in 17 (28%) patients. Also, of the 63 patients seen by genetics, 45 patients underwent genetic testing. The germline testing identified a mismatch repair gene mutation in eight (18%) of the 45 patients: six patients with abnormal immunohistochemistry analysis, one patient with no loss of staining on immunohistochemistry testing, and one patient who had not had immunohistochemistry studies [13].

There is a consensus that immunohistochemistry is a good screening test. However, all the investigators have observed negative immunohistochemistry results in patients who had a mismatch repair gene germline mutation (Table 2) [10,12,13,18,19]. Most of the researchers suggested genetics referral and additional evaluation when the immunohistochemistry analysis demonstrates loss of gene expression based on staining absence [12,13,18]. Indeed, one of the group of investigators commented that the standard for diagnosing Muir-Torre syndrome is germline genetic testing [10].

Microsatellite instability testing

Microsatellite instability assessment of visceral tumors from individuals suspected to have Lynch syndrome or sebaceous neoplasms from patients in whom the diagnosis of Muir-Torre syndrome is being considered utilizes the Bethesda markers recommended by the National Cancer Institute in the United States of America. The five markers consist of three dinucleotide repeats and two mononucleotide tracts. A positive test (microsatellite instability-high) results when microsatellite instability is detected in any two of the five markers; this indicates not only a loss of mismatch repair but also the possibility of Lynch syndrome. In contrast, normal mismatch repair and the absence of Lynch syndrome are indicated by a negative test (microsatellite instability-low) [5,8,9,11,18].

Microsatellite instability is a polymerase chain reaction-based test. Testing for microsatellite instability does not provide specific genomic aberration, and it is a time-consuming technique to perform. However, a microsatellite instability assay that requires less technical time with faster results has recently been developed [9]. Many pathology laboratories, when screening colorectal carcinoma or endometrial cancer for a potential association with Lynch syndrome, simultaneously perform immunohistochemistry analysis and microsatellite instability testing; if the results of one or both studies are positive, patients are often referred for genetic evaluation and/or germline testing [8,9,11,18].

Discordant microsatellite instability test results with immunohistochemistry analysis or germline evaluation or both have been observed by several investigators. Usha et al. described a 43-year-old female with endometrial cancer-associated Lynch syndrome; the screening tests of her tumor demonstrated negative staining for MSH6 by immunohistochemistry (suggesting the possibility of Lynch syndrome), microsatellite instability-low result (associated with a decreased likelihood of Lynch syndrome), and a germline mutation R1331X in the MSH6 gene (which established the diagnosis of Lynch syndrome). Hence, the negative microsatellite instability testing was discordant with both the immunohistochemistry analysis and germline evaluation [8].

Favre et al. studied two polymerase chain reaction-based microsatellite instability testing platforms and compared the results to those obtained using immunohistochemistry analysis of 37 sebaceous neoplasms and two keratoacanthomas. One platform (referred to as pentaplex polymerase chain reaction) used five mononucleotide repeat microsatellite targets; the other platform (referred to as a seven-sequence microsatellite instability assay) performed polymerase chain reaction amplification of seven novel repeat microsatellite sequences followed by high-resolution melting curve analysis. The investigators required at least two markers to be mutated to establish microsatellite instability [9].

The results of immunohistochemistry analysis demonstrated a loss of mismatch repair gene expression for 29 of the 39 tumors that were tested. Concordant results for immunohistochemistry analysis and pentaplex polymerase chain reaction were only observed in 31 of 39 (79%) tumors. In addition, the pentaplex polymerase chain reaction only demonstrated microsatellite instability for 21 of 29 (72%) tumors that had loss of mismatch repair gene expression; hence, the pentaplex polymerase chain reaction had eight (28%) false-negative cases [9].

The seven-sequence microsatellite instability assay had superior results compared to the pentaplex polymerase chain reaction, yet the assay obtained false-negative results in three of 29 (10%) cases. Specifically, 36 of 39 (92%) tumors had concordant immunohistochemistry and microsatellite instability results. However, the seven-sequence microsatellite instability assay was positive for only 26 of 29 (90%) tumors that immunohistochemistry analysis showed a loss of mismatch repair gene expression [9].

Aziz et al. investigated skin tumors in Lynch syndrome patients; the tumors included both Muir-Torre syndrome-associated sebaceous neoplasms and other benign or malignant cutaneous lesions. The study was a retrospective systematic review of the literature that included 278 lesions from 171 patients with molecular confirmation of Lynch syndrome by germline testing that were evaluated by immunohistochemistry or microsatellite instability or both. Microsatellite instability was demonstrated on 103 of 114 (90%) germline-positive Lynch syndrome tumors that were evaluated with this testing. False-negative results of microsatellite stability were observed in 11 of 114 (10%) lesions tested for microsatellite instability; these included not only three Muir-Torre syndrome-related sebaceous neoplasms such as sebaceous adenoma (two) and sebaceous carcinoma (one) but also eight other tumors such as squamous cell carcinoma (six), keratoacanthoma (one), and intradermal nevus (one) [18].

In summary, several studies have demonstrated that the polymerase chain reaction-based microsatellite instability assays result in false-negative results compared to either immunohistochemistry analysis and/or germline testing. Microsatellite instability testing is still used as a screening test, in conjunction with immunohistochemistry analysis, to determine if patients with Lynch syndrome-associated tumors such as colorectal cancer and endometrial carcinoma should be sent for genetic evaluation or germline testing or both. However, the role of microsatellite instability testing of Muir-Torre syndrome-related sebaceous neoplasms remains to be established [9,18].

Next-generation sequencing

Next-generation sequencing is an investigative technique that can be used to define the genomic mutational landscape of a tumor. After determining a cancer’s genetic aberrations, therapy targeted to the specific gene abnormality can be initiated. Although this analysis is not often used in the evaluation of Muir-Torre syndrome-associated sebaceous neoplasm to establish the diagnosis of Lynch syndrome, investigators have evaluated sebaceous skin tumors with next-generation sequencing to determine clinically actionable genes [14,17].

Tetzlaff et al. evaluated 27 sebaceous carcinomas (which included 18 primary ocular tumors, five metastatic ocular tumors, and four primary extraocular tumors) from 20 patients (which included 16 primary ocular tumors, of whom three also had metastatic ocular tumors, and four primary extraocular tumors) by performing whole-exome next-generation sequencing of 409 cancer-associated genes. Mutations in potentially clinically actionable genes were discovered in 14 of 27 (52%) sebaceous carcinomas from nine of 20 (45%) patients (including six of 16 patients with a primary ocular tumor and three of four patients with an extraocular tumor). None of the ocular tumors had mismatch repair gene mutations; however, two of four patients with extraocular tumors had somatic mutations of either MLH1 or MSH2, and these observations were concordant with the results of both immunohistochemistry analysis and microsatellite instability testing [17].

Simic et al. recently studied the clinical and molecular features of 11 skin neoplasms (including nine sebaceous tumors, one melanoma, and one squamous cell carcinoma) in seven patients with Muir-Torre syndrome by performing next-generation sequencing of 161 cancer driver genes. Mismatch repair gene mutations were identified in the sebaceous neoplasms of all the patients. Six patients had a mutation in the MSH2 gene, and one patient had a mutation in the MLH1 gene [14].

Mutations in neurogenic locus notch homolog protein 1 preprotein (NOTCH1 gene) and neurogenic locus notch homolog protein 2 preprotein (NOTCH2 gene) were each present in four sebaceous neoplasms. One patient had two sebaceous neoplasms with mutations of both the NOTCH1 gene and NOTCH2 gene and a third sebaceous neoplasm with a NOTCH2 gene mutation. Another patient had a sebaceous neoplasm with mutations of both the NOTCH1 gene and the NOTCH2 gene. A third patient had sebaceous neoplasm with a NOTCH1 gene mutation. Mutations in 16 other genes were also noted only once in one of the nine sebaceous neoplasms [14].

In summary, precision oncology can utilize next-generation sequencing of Lynch syndrome-related malignancies to discover potential clinically actionable genes for targeted cancer therapy. Next-generation sequencing is also able to reliably assess the genomic aberrations of Muir-Torre syndrome-associated sebaceous neoplasms and establish whether mutations of mismatch repair genes are present. However, similar to immunohistochemistry and microsatellite instability evaluation, next-generation sequencing of tumor tissue cannot determine whether the observed gene mutations are somatic or germline [14,17].

Mayo Muir-Torre syndrome risk score

A group of researchers proposed an algorithm in 2014, referred to as the Mayo Muir-Torre syndrome risk score, as a screening tool to identify which patients with at least one sebaceous skin neoplasm (such as a sebaceous adenoma, sebaceous epithelioma/sebaceoma, and sebaceous carcinoma) were most likely to have Muir-Torre syndrome and should therefore have germline genetic testing for mismatch repair genes (Table 3) [3]. As part of the algorithm, the investigators defined colorectal, endometrial, ovarian, small bowel, urinary tract (renal pelvis and ureter), and biliary tract (gallbladder and bile ducts) cancers as Lynch syndrome-related malignancies. However, assessment of the sebaceous neoplasm with either immunohistochemistry analysis or microsatellite instability testing or both was not included in the algorithm [3].

**Table 3:** Result of Mayo Muir-Torre syndrome risk score algorithm applied to men in this report Abbreviations: C, case; Cor, correlation; dx, diagnosis; GlM, germline mutation; LSrC, Lynch syndrome-related cancer; MLH1, MutL Homolog 1 gene; MMTS, Mayo Muir-Torre syndrome risk score; MTS, Muir-Torre syndrome; PHx, personal history; PMS2, PMS1 Homolog 2, mismatch repair system component gene; Scr, score; SN, sebaceous neoplasms; TND, testing not done; Unk, unknown; yrs, years, #, total number; &, and; =, equals ^a^SN includes adenomas, epitheliomas/sebaceomas, and carcinomas. SN dx age (yrs): 60 or older, scr = 0; younger than 60, has scr = 1. ^b^SN #: one SN, scr = 0; two or more SN, scr = 2. ^c^LSrC includes colorectal, endometrial, ovarian, small bowel, urinary tract (renal pelvis and ureter), and biliary tract (gallbladder and bile ducts) cancers. ^d^Any LSrC-PHx: no, scr = 0; yes, scr = 1. ^e^Any LSrC-FHx: no, scr = 0; yes, scr = 1. ^f^The total scr (MMTS) can range from 0 to 5; it is the sum of the scrs for the four variables. There is a 100% sensitivity and an 81% specificity for predicting a GlM in a Lynch syndrome mismatch repair gene if the MMTS is two or greater. MTS was diagnosed in 12 of 20 patients with a scr of 2 and in 28 of 29 patients with a scr of 3 or more; none of the 39 patients with a scr of 0 or 1 had MTS [3]. ^g^The man had sebaceous carcinoma. ^h^The man had sebaceous epithelioma. ^i^The man’s maternal uncle had colon cancer. ^j^The man had sebaceous adenoma. ^k^The man had colon cancer. ^l^The man’s mother had primary colorectal cancer twice and cancer of her pancreas and liver; his brother had kidney and rectal cancer.

C	Age at SN dx (yrs)	Scr^a^	# of SN	Scr^b^	PHx of LSrC^c^	Scr^d^	FHx of LSrC^c^	Scr^e^	Total Scr^f^	GlM	Cor of MMTS & GlM
1	67	0	1^g^	0	No	0	No	0	0	TND	Unk
2	74	0	1^h^	0	No	0	Yes^i^	1	1	PMS2	No
3	59	1	1^j^	0	Yes^k^	1	Yes^l^	1	3	MLH1	Yes

The study, for which the Mayo Muir-Torre syndrome risk score was derived, included 89 patients referred by cancer-specific genetic counselors. The Mayo Muir-Torre syndrome risk score (which can range from 0 to 5) was determined by assigning a numeric score to each of four categories: (1) sebaceous neoplasm diagnosis age (60 years older = 0 and younger than 60 years = 1), (2) sebaceous neoplasm total number (one neoplasm = 0 and two or more neoplasms = 2), and whether any Lynch syndrome-related cancer is present in either (3) the patient (no personal history = 0 and a positive personal history = 1) or (4) the patient’s family (no family history = 0 and a positive family history = 1). The researchers found that their algorithm had a 100% sensitivity and an 81% specificity for predicting a Lynch syndrome-associated mismatch repair gene germline mutation if the Mayo Muir-Torre syndrome risk score was 2 or more. However, the investigators emphasized that their risk score algorithm still needed to be validated by other researchers using an independent group of patients and that they were not certain if the risk score algorithm would be valid in a patient with preservation of mismatch repair gene expression demonstrated by positive staining on immunohistochemistry analysis of their sebaceous neoplasm [3].

The Mayo Muir-Torre syndrome risk score provided a false-negative result for the 74-year-old man in this report (Table 3). Although his immunohistochemistry analysis showed a loss of PMS2 gene expression, his risk score was only 1. However, germline testing for a mismatch repair gene mutation confirmed the suspected diagnosis of prior PMS2-associated Lynch syndrome in a man with newly presenting germline-confirmed PMS2-associated Muir-Torre syndrome.

Germline testing

Lee et al. did a retrospective study of patients with sebaceous neoplasms including sebaceous adenomas, sebaceomas, and sebaceous carcinomas that noted the frequency of a prior, concurrent, or subsequent visceral tumor. They observed an increased incidence of internal malignancy in patients with one or more Muir-Torre syndrome-associated sebaceous neoplasms; 19 of 85 (22%) patients had at least one visceral cancer. They also noted a significant rate of false-negative immunohistochemistry analysis results in the sebaceous neoplasms of the cancer patients; indeed, seven of 16 (44%) of these patients who had immunohistochemistry testing of their sebaceous neoplasm did not demonstrate any loss of mismatch repair gene expression [20].

The United Kingdom National Institute for Health and Care Excellence recommends germline evaluation of mismatch repair genes to screen for Lynch syndrome in all patients with colorectal carcinoma even though the expected yield is only 3%. Similar to Lee et al., Gallon et al. also observed an increased rate of cancer in patients with Muir-Torre syndrome-associated sebaceous neoplasms; specifically, they noted that the incidence of Lynch syndrome diagnosed by germline-confirmed mismatch repair gene mutations was 33.3% in patients with Muir-Torre syndrome-associated sebaceous neoplasms [15,20]. Therefore, Gallon et al. proclaimed that Muir-Torre syndrome-associated sebaceous neoplasms are a prototypical class of cutaneous tumor for universal germline genetic testing [15].

Germline testing of the mismatch repair genes, prompted by his mother having not only two primary colorectal cancers but also pancreas and liver cancer, discovered an MLH1 gene mutation in the 59-year-old man in this report whose early detection of colon carcinoma resulted from his regularly scheduled colonoscopies for cancer screening. It was unexpected that the immunohistochemistry analysis of his sebaceous adenoma showed normal staining for MLH1. In addition, had mismatch repair gene germline testing not been performed on the 74-year-old man in this report, his diagnosis of PMS2-associated Muir-Torre syndrome would not have been established. Germline testing of his son, who had acute myelogenous leukemia, also demonstrated PMS2-associated Lynch syndrome. The 67-year-old man in this report with loss of MSH2 and MSH6 staining on immunohistochemistry analysis of his extraocular sebaceous carcinoma was sent for oncology and genetics evaluations; the referral laboratory commented that adequate tumor tissue was not available for next-generation sequencing, and he subsequently declined germline testing since his Mayo Muir-Torre syndrome risk score was less than 2.

We concur with the recommendation by Gallon et al. that germline genetic testing for mismatch repair genes should be done in all patients with Muir-Torre syndrome-associated sebaceous neoplasms to identify individuals at risk for Lynch syndrome [15]. Indeed, all the studies on immunohistochemistry analysis and microsatellite instability evaluation in Muir-Torre syndrome-related sebaceous tumors have demonstrated false-negative test results. In addition, next-generation sequencing of tumor tissue is not able to differentiate a somatic gene mutation from a germline genomic aberration; sequencing of blood, saliva, or normal skin biopsy is needed to demonstrate a germline mismatch repair gene mutation. Also, based solely on medical economics, mismatch repair gene germline testing as part of the initial evaluation of a patient with even a single new Muir-Torre syndrome-associated sebaceous neoplasm is cost-effective; if a germline mutation is discovered, the patient can be referred to genetic counseling for not only recommendations regarding initial and future cancer screening but also germline testing and, if indicated, subsequent cancer screening of family members.

## Conclusions

Muir-Torre syndrome is a subtype of Lynch syndrome characterized by the development of at least one syndrome-related sebaceous neoplasm such as an adenoma, epithelioma, or carcinoma and a systemic malignancy. The genodermatosis has an autosomal dominant mode of inheritance; however, it can occur as a non-inherited sporadic condition. The syndrome results from the microsatellite instability caused by a germline mutation of MLH1, MSH2, MSH6, and/or PMS2 mismatch repair genes. Genetic counseling and germline evaluation for mismatch repair gene mutations has been previously based on the abnormal results of immunohistochemistry analysis or microsatellite instability testing or both performed on Muir-Torre syndrome-related sebaceous neoplasms or visceral cancers such as colorectal and endometrial carcinoma.

Three men with new-onset Muir-Torre syndrome-related sebaceous neoplasm are described. The first patient was a 67-year-old male who had a sebaceous carcinoma. Immunohistochemistry analysis of the sebaceous carcinoma showed a loss of expression for MSH2 gene and MSH6 gene. He had no personal or family history of cancer. His Mayo Muir-Torre syndrome risk score was 0, and he declined germline testing. The second patient was a 74-year-old male who had a sebaceous epithelioma. Immunohistochemistry analysis of the sebaceous epithelioma showed a loss of PMS2 gene expression. He had no personal history of Lynch syndrome-related cancer; however, he had a family history of colon cancer. His Mayo Muir-Torre syndrome risk score was only 1; germline testing is usually not recommended for individuals with scores less than 2. However, germline testing was performed and confirmed a diagnosis of PMS2-associated Muir-Torre syndrome. The third patient was a 59-year-old male who had an antecedent diagnosis of germline-confirmed MLH1-associated Lynch syndrome and a newly diagnosed sebaceous adenoma. Immunohistochemistry analysis for mismatch repair gene expression of his sebaceous adenoma was performed; the observed normal results were unexpected.

In summary, the incidence of false-negative results with immunohistochemistry analysis and microsatellite instability testing is significant; in addition, neither these tests nor next-generation sequencing of tumor tissue can definitively differentiate an inherited germline mutation from an acquired somatic genomic aberration of the mismatch repair genes. Also, similar to the 74-year-old male described in this report, the Mayo Muir-Torre syndrome risk score algorithm is not always reliable for determining which patient with a new Muir-Torre syndrome-related sebaceous neoplasm should have germline testing. Therefore, we propose that germline testing (on normal elements such as blood, saliva, or a normal skin biopsy) for a mismatch repair gene mutation should be the initial evaluation of a patient with even a single new Muir-Torre syndrome-associated sebaceous neoplasm. A positive germline aberration will not only establish that an inherited syndrome-related mutation of a mismatch repair gene is present but also that the patient should be referred for genetic counseling regarding initial and future cancer screening recommendations and that the patient’s family members should have germline testing and cancer screening, if indicated.
